# Supplementary material for: The Role of the Environment in Dynamics of Antibiotic Resistance in Humans and Animals: A Modelling Study
Source: Antibiotics (Basel). 2022 Oct 5;11(10):1361. doi: 10.3390/antibiotics11101361 (PMC9598675; doi:10.3390/antibiotics11101361)
Supplement: Supplementary file 1 [file antibiotics-11-01361-s001.zip › antibiotics-1813534-supplementary.pdf]

Appendix for manuscript: The role of the environment in transmission of antimicrobial resistance between humans and animals

Table S1: Parameter definitions

Parameter	Definition and units
β_{HH}	Per capita rate at which humans acquire antibiotic resistant bacteria as a result of exposure to other humans harbouring resistant bacteria per time step
β_{AA}	Per capita rate at which animals acquire antibiotic resistant bacteria as a result of exposure to other animals harbouring resistant bacteria per time step
β_{AH}	Per capita rate at which humans acquire antibiotic resistant bacteria as a result of exposure to animals harbouring resistant bacteria per time step
β_{HA}	Per capita rate at which animals acquire antibiotic resistant bacteria as a result of exposure to humans harbouring resistant bacteria per time step
β_{HE}	Per environmental unit rate* at which the environment acquires resistant bacteria as a result of exposure to humans harbouring resistant bacteria per time step
β_{EH}	Per capita rate at which humans acquire antibiotic resistant bacteria as a result of exposure to environmental units harbouring resistant bacteria per time step
β_{AE}	Per environmental unit rate* at which the environment acquires resistant bacteria as a result of exposure to animals harbouring resistant bacteria per time step
β_{EA}	Per capita rate at which animals acquire antibiotic resistant bacteria as a result of exposure to environmental units harbouring resistant bacteria per time step
Λ_{H}	Per capita rate at which humans acquire antibiotic resistant bacteria as a result of direct exposure to antibiotics per time step
Λ_{A}	Per capita rate at which animals acquire antibiotic resistant bacteria as a result of direct exposure to antibiotics per time step
γ_H	Proportion of Λ_{H} that reaches the environment as antibiotics (a scalar parameter)
γ_A	Proportion of Λ_{A} that reaches the environment as antibiotics (a scalar parameter)
$\gamma_H\Lambda_H$	Per environmental unit rate* at which the environment acquires resistant bacteria as a result of exposure to a proportion of antibiotics given to humans per time step
μ_H	Per capita rate at which humans with resistant bacteria revert to have only sensitive bacteria per time step
μ_A	Per capita rate at which animals with resistant bacteria revert to have only sensitive bacteria per time step
μ_E	Per environmental unit rate* at which environmental units harbouring resistant bacteria revert to having only sensitive bacteria per time step

Appendix for manuscript: The role of the environment in transmission of antimicrobial resistance between humans and animals

Table S2: Parameter values

Table S2A: Transmission coefficients

Unbounded model

**Parameter**	**Balanced**	**Balanced (low β_{HA})**	**Environment-driven**	**Animal-driven**	**Human-driven**
β_{HH}	0.074	0.074	0.001	0.001	0.202
β_{AA}	0.074	0.074	0.001	0.202	0.001
β_{HA}	0.074	0.0007	0.001	0.001	0.202
β_{AH}	0.074	0.074	0.001	0.202	0.001
β_{EH}	0.074	0.074	0.142	0.001	0.001
β_{EA}	0.074	0.074	0.142	0.001	0.001
β_{HE}	0.074	0.074	0.142	0.001	0.202
β_{AE}	0.074	0.074	0.142	0.202	0.001

Bounded model

**Parameter**	**Balanced**	**Balanced (low β_{HA})**	**Environment-driven**	**Animal-driven**	**Human-driven**
β_{HH}	0.081	0.081	0.001	0.001	0.202
β_{AA}	0.081	0.081	0.001	0.202	0.001
β_{HA}	0.081	0.0008	0.001	0.001	0.202
β_{AH}	0.081	0.081	0.001	0.202	0.001
β_{EH}	0.081	0.081	0.231	0.001	0.001
β_{EA}	0.081	0.081	0.231	0.001	0.001
β_{HE}	0.081	0.081	0.231	0.001	0.202
β_{AE}	0.081	0.081	0.231	0.202	0.001

Table S2B: Other parameters

**Parameter**	**Value**	
	**Fig 1. B**	**Fig 2. And 3.**
Λ_H	0.1	Beta(1.7, 15.3) (mean 0.1)
Λ_A	0.1	No intervention: 0.1 or U(0.000001,1.); intervention: 0.0.
γ_H	0.001	0.001
γ_A	0.001	0.001
μ_H	0.1	Beta(1.7, 15.3) (mean 0.1)
μ_A	0.1	0.1
μ_E	0.2	Beta(3, 12) (mean 0.2)

Additional methods information

Methods for finding transmission parameter coefficients

Transmission parameters were chosen by the following method: some parameters were fixed (p_f) whilst the transmission parameters of interest varied (p_v) to reach a human resistance level of 54.1% (rate of ampicillin resistance in clinical isolates of *E. coli* in the EU in 2020, based on EARS-Net data[1]):

$$\min_{p_v \in (0,1)} \left| 0.71 - f(p_v, p_f) \right|$$

For example, for the human-driven transmission scenario, rates of transfer of resistance from humans to any other population were varied ($\beta_{HH} = \beta_{HA} = \beta_{HE}$), and all other transmission parameter values were fixed at a low value, 0.001.

Model timesteps

To ensure equilibrium values were obtained for all experiments, we initially numerically solved the model to 500 timesteps, and if there was a difference of more than 0.0000001 between the R_H values for the final two timesteps, we solved to 10,000 timesteps.

Appendix for manuscript: The role of the environment in transmission of antimicrobial resistance between humans and animals

Figure S1: Trajectory plot of the fraction of human and animal populations carrying resistant bacteria (R_H, R_E), and the amount of resistant material in the environment (R_E). For bounded environment model (left) and unbounded (right). Uses the balanced transmission scenario.

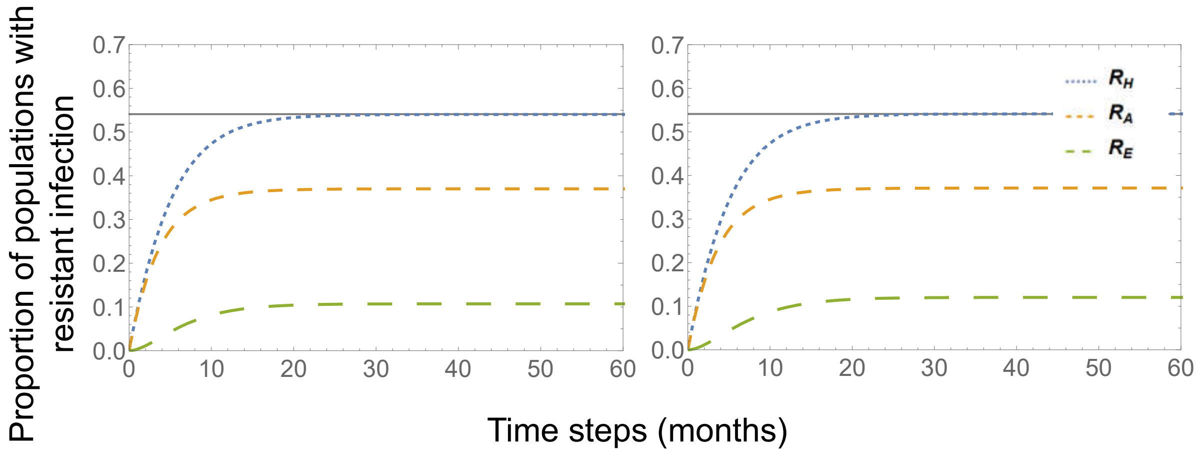

Figure S2: Heatmaps of the impact of reducing Λ_A, for different pre-intervention levels of Λ_A (Y axis) and β_{EH} (X axis), in all transmission scenarios. Plot A) uses the bounded, and plot B) uses the unbounded versions of the model.

A

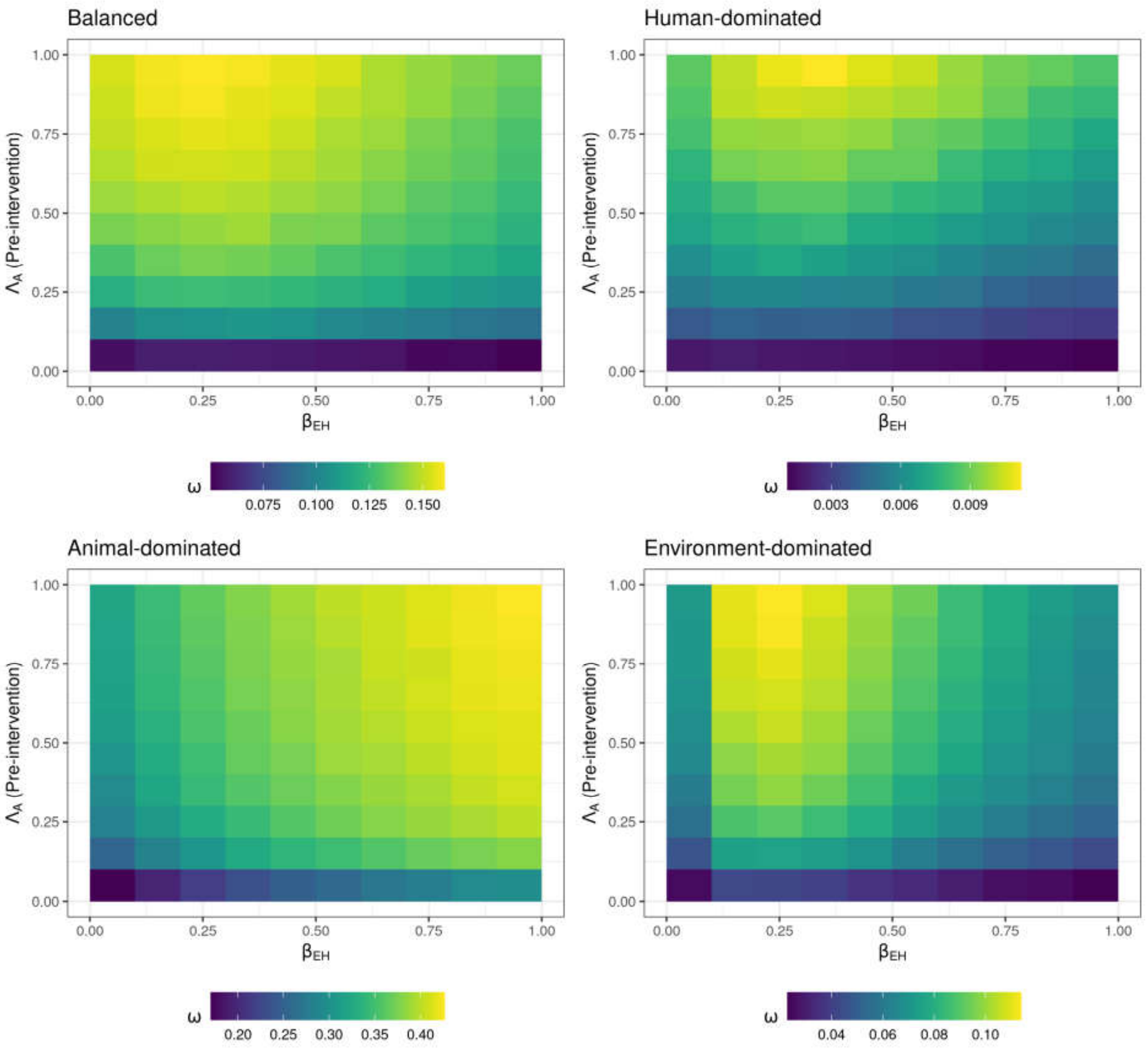

B

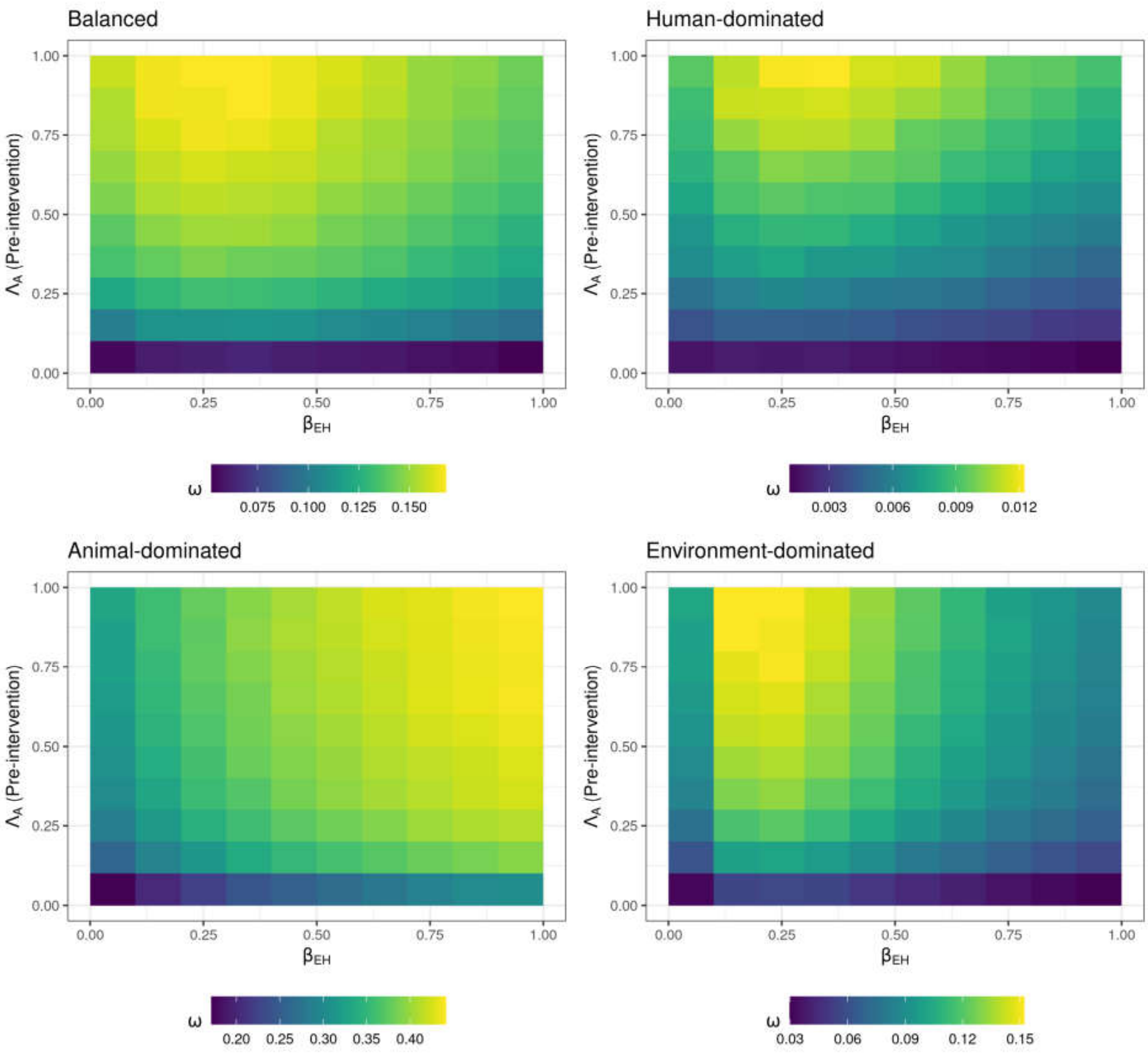

Appendix references

1. WHO Regional Office for Europe/European Centre for Disease Prevention and Control. *Antimicrobial Resistance Surveillance in Europe 2022 - 2020 Data*. WHO Regional Office for Europe; 2022.
